# Lessons from the SWITCH trial: changing glucocorticoids in the management of metastatic castration-resistant prostate cancer (mCRPC)

**DOI:** 10.1038/s41416-018-0239-y

**Published:** 2018-10-22

**Authors:** Min Yuen Teo, Howard I. Scher

**Affiliations:** 1 Genitourinary Oncology Service, Department of Medicine, Memorial Sloan Kettering Cancer Center, New York, NY USA; 2 Department of Medicine, Weill Cornell Medical College, New York, NY USA

## Abstract

Abiraterone acetate plus prednisone is a standard treatment option for mCRPC. The phase II SWITCH trial showed that further prostate-specific antigen (PSA) responses can be obtained in a subset of patients when prednisone was switched to dexamethasone at progression. Here, we discuss the potential underlying mechanisms, including the activation of glucocorticoid receptors (GR) in progressive mCRPC and the implications for clinical practice.

Advanced prostate cancer is an androgen-driven disease for which androgen deprivation therapy (ADT) with gonadotropin analogues is a first-line standard of care. The response can be dramatic as seen by the declines in PSA, shrinkage of tumour and resolution of symptoms. ADT is not curative, and after variable periods of time the disease evolves to a castration-resistant state, the lethal form of the disease, largely due to the reactivating alterations in the androgen receptor (AR) signalling axis, including overexpression of the androgen biosynthetic machinery leading to an increase in intratumoural androgens and AR overexpression. Here, approved life-prolonging therapies include next-generation hormonal agents targeting these AR alterations (abiraterone acetate plus prednisone [A + P] and anti-androgens enzalutamide and apalutamide), cytotoxic chemotherapy (docetaxel and cabazitaxel), immunotherapy (sipleucel-T) and alpha-emitting bone-targeted radionuclides (radium-223). Prednisone alone can palliate symptoms, but has not been proven to prolong life.

In this issue, Romero-Laorden and colleagues report on the phase II SWITCH trial, in which patients with mCRPC who were progressing on A + P (5 mg twice daily) responded when prednisone was stopped and dexamethasone (0.5 mg daily) was started: a glucocorticoid switch.^1^ The rationale was based on a retrospective evaluation of 30 mCRPC patients treated with A + P who received dexamethasone upon progression, in which ≥30% declines in PSA that persisted for a median of 20.6 weeks were observed.^2^ Enrolment on the SWITCH trial was limited to patients with PSA progression alone or PSA progression with ‘modest’ changes in imaging, defined as three or fewer new asymptomatic lesions on bone scan, absence of new soft-tissue disease and a <40% increase in the size of pre-existing target lesions per RECIST 1.1 criteria.^1^ Overall, 26 patients were enrolled, 14 of whom (53.8%) were docetaxel-naïve and 12 (46.2%) had radiographic evidence of progression. Consistent with the prior observations, a ≥30% fall in PSA that was maintained for ≥6 weeks was observed in 12 (46.2%) cases and a ≥50% fall in PSA for ≥12 weeks in eight (34.6%) cases following the switch to dexamethasone. The observed adverse events were consistent with the known effects of glucocorticoids including muscle weakness, hypertension and hyperglycaemia, none of which were grade 3 or greater.

Corticosteroids, and prednisone in particular, are an integral component of many treatment regimens for mCRPC, but for different reasons. First is the recognition of benefit when given as monotherapy,^3^ and that the survival benefit shown for both docetaxel and cabazitaxel was in combination with prednisone.^4,5^ Second is their use to reduce the side effects associated with mineralocorticoid excess resulting from inhibition of Cyp17, a key enzyme in androgen synthesis, from agents such as abiraterone acetate.^6^ Here, it is important to recognise that the effects of individual glucocorticoids differ both in efficacy and the context in which they have been used. In one single-centre phase II study in chemotherapy-naïve mCRPC, patients were randomised to prednisone 5 mg twice a day, dexamethasone 0.5 mg daily or intermittent dexamethasone at 8 mg for 3 days every 3 weeks. The results showed a higher PSA response rate and longer time to PSA progression in the continuous dexamethasone arm relative to prednisone (41 vs. 22% and 9.7 vs. 5.1 months, respectively.^7^) Intermittent dexamethasone was inactive. In the phase I/II trial of abiraterone acetate monotherapy, the addition of dexamethasone at the time of progression led to an observable PSA response in up to a third of patients.^6^

Several hypotheses have been proposed to explain the responses observed in the earlier SWITCH study^2^ including, but not limited to: [I] the emergence or selection of cells with secondary AR mutations that are activated by prednisone but not dexamethasone;^8^ [II] a differential downstream transcriptional effect of 0.5 mg of dexamethasone versus 10 mg of prednisone in cells overexpressing the GR as a bypass mechanism to AR inhibition and [III] secondary activation of the mineralocorticoid receptor, which has higher affinity for prednisone.^9^

Dexamethasone and prednisone harbour differences in structure, which in other settings might be associated with differences in receptor binding and affinities and potency, resulting in different pharmacodynamic properties.^10^ Moreover, 10 mg of prednisone is equipotent with 1.6 mg of dexamethasome, making the SWITCH trial more than a simple switch. GR and AR are similar in structure and share similar target gene responses, and in vivo studies have shown that resistance to AR-targeting therapies can occur through overexpression of GR that results in transcriptional activation of a subset of AR targets, in addition to GR-specific targets. This observation was supported by the clinical studies of GR expression in mCRPC biopsy samples^11^ and after enzalutamide exposure.^12^ In the latter, 27 patients had a baseline and an 8-week bone marrow biopsy after starting enzalutamide analysed based on PSA response and clinical benefit. Baseline GR expression by immunohistochemistry is low (range: 3–8%), GR expression at 8 weeks was significantly higher among the patients who did not derive prolonged clinical benefit from enzalutamide (29 vs. 10%).^12^ This represents some of the first clinical evidence that, in a subset of patients, AR inhibition drives GR overexpression, which contributes to subsequent resistance to AR-directed therapy. Supporting this hypothesis further is the observation that modulation of the GR can delay growth and CRPC progression in pre-clinical models.^13^

The phase II SWITCH trial was well designed and executed, but as the authors correctly pointed out, it remains a proof-of-concept study with limited generalisability. One reason is due to differences in the patient populations in SWITCH and the pre- (COUGAR-AA-302) and post- (COUGAR-AA-301) registration trials as shown by shorter median biochemical progression-free survival times prior to the switch of 5.4, 11.1^14^ and 10.2 months, respectively.^15^ The ≥50% PSA decline rate A + P in SWITCH was only 42 and 21% for chemotherapy-naïve and docetaxel-exposed patients, respectively, compared with 62 and 38% in the corresponding registration trials. Uncertain as well is when ‘switch’ should occur when the signs of disease progression are manifested. Notable here is that the second Prostate Cancer Working Group (PCWG2) advised caution when a rising PSA was the sole manifestation of progression in the absence of other signs, and PCWG3, the most recent iteration, extended this further by introducing the concept of ‘no longer clinically benefiting’ (NLCB): the decision of when to change the therapy in the setting of a slowly rising PSA or small-volume radiographic progression after an initial response, recognising that the biological heterogeneity of individual metastatic lesions is such that some lesions may continue to respond while others progress.^16^

PCWG3 also noted that the detection of a molecular alteration putatively validated in preclinical model systems to confer resistance and drive tumour growth does not necessarily mean it is contributing to tumour growth in a patient at a given point in time. In SWITCH, a T878A-point mutation in the *AR* gene was found in six (23%) patients and associated with A + P resistance,^8^ of whom three had a ≥30% and two a ≥50% PSA decline that met the defined primary endpoint after the SWITCH, although, as the authors pointed out, the duration was short. Impotant here is that *AR* mutations can be detected to varying degress in over 7% of newly diagnosed and progressing mCRPC,^17^ making it difficult to determine their contribution to tumour growth and duration of disease following a therapy directed to AR or GR signalling. More important is the need for additional evidence of benefit beyond PSA declines, particularly those that are not durable. As an example, when the NLCB stopping rule was applied in the phase 2 apalutamide study, in a post-abiraterone cohort, 43% of patients remained on therapy for months or more before a clinically indicated change in treatment was needed, despite a protocol-defined PSA response rate of 22%.^18^

The real question is how the findings of the SWITCH study will impact the day-to-day clinical practice? Here, a change to a drug of the same class, ‘patient neutral’ in terms of how it is administered and adverse event profile, provided durable benefit to a subset of patients. In the recent Advanced Prostate Cancer Consensus Conference, 71% (35 of 49) of the panellists supported the steroid switch as reasonable for some asymptomatic mCPRP patients with a rising PSA while on abiraterone plus prednisone. We concur for selected patients with no or limited radiographic progression recognising that it will not benefit all patients. The eligibility was likely based on the assumption that patients with rapid progression would be at greater risk from the switch and not respond. It may be, as with other effective targeted therapies, that the more clinically aggressive tumours may be more sensitive to the SWITCH. Unknown as well, now that ADT in combination with A + P is a new standard of care for non-castrate metastatic disease based on the MRC STAMPEDE and LATITUDE trials, is whether a change from prednisone to decadron in this context would provide results similar to what was seen in SWITCH.^19^

The study is an important contribution to the growing literature on the role of corticosteroids and GR in CRPC, now an increasing focus of clinical investigation in several ongoing clinical trials, including phase I/II studies of enzalutamide plus mifepristone (NCT02012296) and enzalutamide plus CORT125281, a selective GR antagonist (NCT03437941). It also illustrates the need for a clear understanding of who might benefit, be it on clinical criteria or biologic profiling, and when it should be considered to ensure maximal patient benefit. Faced with rising drug costs and limited resources, simple manoeuvres such as this, similar to the anti-androgen withdrawal syndrome described in the 1990s,^20^ do have an important impact.
